# Supercooling Behavior of 2-Amino-2-methyl-1,3-propanediol for Thermal Energy Storage

**DOI:** 10.3390/molecules30102206

**Published:** 2025-05-18

**Authors:** Xuelian Wang, Jin Bai, Xian Zhang, Xiaobo Shen, Zhengrong Xia, Haijun Yu

**Affiliations:** 1 School of Electronic Engineering, Huainan Normal University, Huainan 232038, China; wangxuelian@mail.ustc.edu.cn (X.W.); shenxb@hnnu.edu.cn (X.S.); zhengrongxia@hnnu.edu.cn (Z.X.); haijun20030@163.com (H.Y.); 2 Key Laboratory of Materials Physics, Institute of Solid State Physics, The Hefei Institutes of Physical Science (HFIPS), Chinese Academy of Sciences, Hefei 230031, China

**Keywords:** AMPD, phase change material, supercooling state, mechanically triggered, thermal management

## Abstract

With the increasing demand for thermal management in electronic devices, highly efficient and controllable phase change materials have attracted significant attention. The compound 2-amino-2-methyl-1,3-propanediol (AMPD), as a solid–solid phase change material, exhibits remarkable supercooling behavior and a high latent heat storage (Δ*H*_endo_ = 247.9 J/g). However, its phase transition kinetics and mechanically triggered properties have not been systematically investigated. In this study, the phase transition behavior of AMPD under different cooling rates and thermal cycling conditions was systematically analyzed using differential scanning calorimetry (DSC). Furthermore, the mechanical triggering characteristic of AMPD under a supercooled state was also studied. The results demonstrated that AMPD can maintain a supercooled state for an extended period, and the exothermic enthalpy change (Δ*H*_exo_) increased by 17.8% (from 154.1 to 181.6 J/g) during thermal cycling. Additionally, mechanical triggering could induce rapid heat release from AMPD, enabling the on-demand regulation of heat utilization. This study revealed that AMPD enables stable supercooling and controllable heat release via thermal or mechanical triggers, offering a novel strategy for tunable solid–solid phase change materials.

## 1. Introduction

The increasing global energy demand and urgent pursuit for carbon neutrality have underlined thermal energy storage technologies as the critical enablers of sustainable energy transitions [1,2]. Phase change materials (PCMs), renowned for their high latent heat capacity and reversible energy storage capabilities, have become indispensable in applications spanning industrial waste heat recovery to advanced microelectronic thermal management systems [3,4]. Among PCMs, solid–solid PCMs stand out for their leakage-free operation and exceptional volumetric stability, making them ideal for precision-critical fields such as aerospace thermal regulation and flexible wearable devices [5,6]. However, the limited availability of high-performance solid–solid PCMs and insufficient understanding about their phase transition kinetics still impede progress toward next-generation thermal management solutions [7].

The compound 2-amino-2-methyl-1,3-propanediol (AMPD) is a multifunctional polyol containing both hydroxyl and amino groups, widely utilized in pharmaceutical synthesis and asymmetric catalysis [8]. Its structural analog, 2-amino-2-methyl-1-propanol (AMP, Δ*H*_exo_ = 134 J/g), corresponding to a glassy-to-ordered crystal transition, has been extensively studied as a solid–solid phase change material due to its supercooling behavior, while the thermal energy storage potential of AMPD remains unexplored despite its superior molecular architecture [9]. The additional hydroxyl group in AMPD strengthens hydrogen-bonding interactions, enhancing structural stability during phase transitions and theoretically optimizing the energy storage capacity [10]. Compared to AMP, AMPD exhibits a 35.5% higher exothermic enthalpy (Δ*H*_exo_ = 181.6 J/g) and enables pressure-independent energy release through mechanical triggering, eliminating the need for the 6.7 MPa external pressure required in plastic crystal systems [11,12,13]. The decoupling from pressure-driven energy release significantly enhances both the safety and operational feasibility. However, the critical issues remain in exploring the thermal storage performance of AMPD. Fundamental thermodynamic parameters, including latent heat, supercooling behavior, and cycling stability, have still not been fully quantified, and its mechanically triggered energy release mechanism requires systematic evaluation to determine its scalability. Addressing these key issues is crucial for realizing AMPD as a potential next-generation PCM for thermal management applications [14,15].

Herein, we present the first comprehensive investigation into the phase transition behavior of AMPD, demonstrating its exceptional supercooling stability, tunable crystallization kinetics, and mechanically triggered heat release. These findings highlight the potential of AMPD as a solid–solid phase change material for thermal energy storage, with the ability to achieve an exothermic enthalpy increase of 17.8% through thermal cycling, and the release of stored energy on demand via mechanical activation. Additionally, the successful design and implementation of AMPD-based waste heat recovery modules further illustrate its practical application in electronic thermal management and off-grid heating scenarios. This work demonstrated that AMPD exhibits dual-controllable supercooling and latent heat release behavior, providing a new model for on-demand solid–solid thermal energy storage.

## 2. Results and Discussion

**Figure 1:**
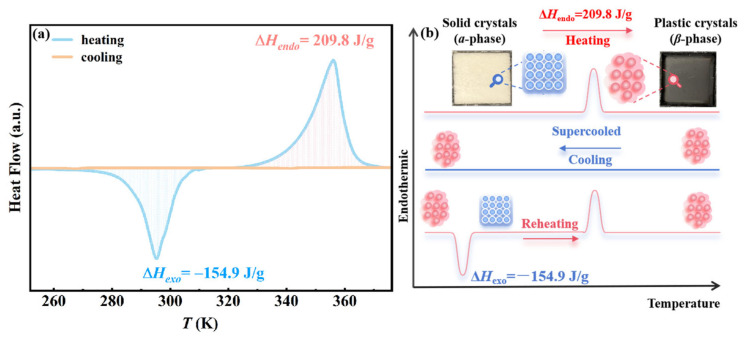
(**a**) The heat flow curve of AMPD as a function of temperature. The yellow curve represents the cooling process, while the blue curve corresponds to the subsequent heating process. Δ*H*_exo_ and Δ*H*_endo_ denote the exothermic and endothermic enthalpy changes, respectively. The negative values of Δ*H*_exo_ indicate heat release. (**b**) Schematic illustration of the structural changes among the ordered *α*-phase, the plastic crystalline *β*-phase, and the metastable supercooled *β*-phase during thermal cycling.

The commercially obtained AMPD exists as a white crystalline solid (*α*-phase) at room temperature. Thermal pretreatment at 360 K induced its transformation into a colorless plastic crystalline state (*β*-phase), enabling the subsequent phase transition process [16]. A sealed aluminum crucible containing 11.42 mg of AMPD was subjected to DSC analysis under controlled cooling and heating rates of 2 K/min. As shown by the yellow curve in Figure 1a, no discernible exothermic crystallization peak was observed during cooling from 360 to 250 K, confirming AMPD retained a supercooled state without spontaneous nucleation. The exceptional supercooling stability originates from its three-dimensional hydrogen-bonding network, formed through synergistic interactions between the hydroxyl (-OH) and amino (-NH_2_) groups, which effectively suppresses molecular rearrangement and crystalline nucleation [17,18]. During the subsequent heating process (blue curve, Figure 1a), AMPD exhibited two distinct phase transitions. The first was an exothermic peak at 290 K (Δ*H*_exo_ = −154.9 J/g), corresponding to the crystallization of the supercooled *β*-phase into the low-temperature *α*-phase. The second was an endothermic peak at 360 K (Δ*H*_endo_ = 209.8 J/g), indicating the phase transition from *α*- to *β*-phase, where AMPD reverts from the crystalline solid to the plastic crystalline state. The structural features and transition pathway are further illustrated in Figure 1b, which provides a visual summary of the reversible phase evolution between the *α*-phase, *β*-phase, and the metastable supercooled state. This phase behavior highlights the dynamic stabilization of both the crystalline and plastic crystal states by the hydrogen-bonding network during thermal energy storage and release [8].

**Figure 2:**
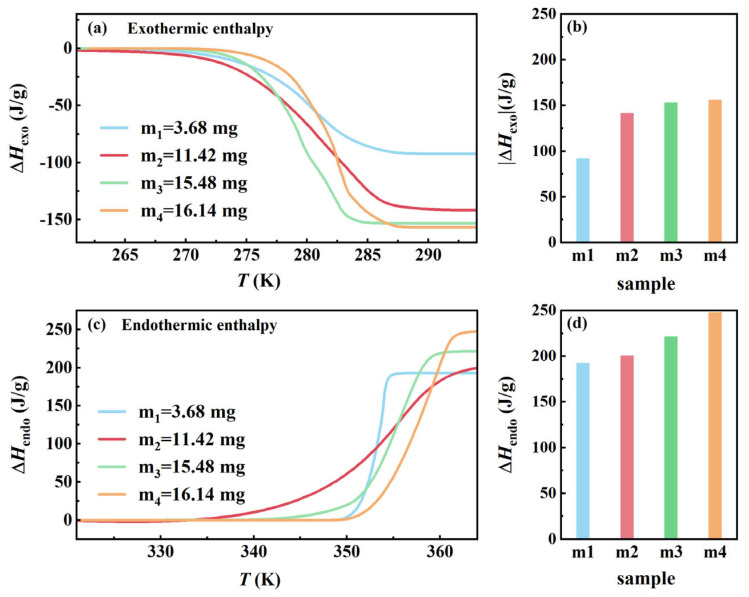
Enthalpy changes during the phase transition of AMPD with varying sample masses. (**a**) Enthalpy changes curves as a function of temperature during the reheating process. (**b**) Variations in exothermic enthalpy change (absolute value) for samples of different masses. (**c**) Enthalpy changes curves as a function of temperature during the heating process. (**d**) Variations in endothermic enthalpy change for samples of different masses.

To investigate the effect of sample mass on the phase transition behavior of AMPD, four different sample masses (*m*_1_ = 3.68 mg, *m*_2_ = 11.42 mg, *m*_3_ = 15.48 mg, and *m*_4_ = 16.14 mg) were subjected to DSC analysis under a uniform heating and cooling rate of 10 K/min. Figure 2a,b present the variations in exothermic enthalpy with temperature and the exothermic enthalpy per unit mass for the samples of different masses during the *β*- to *α*-phase transition. Figure 2c,d show the variations in endothermic enthalpy with temperature, and the endothermic enthalpy per unit mass for the samples of different masses during the *α*- to *β*- phase transition. Larger samples demonstrated progressively higher enthalpy values, culminating in the exothermic enthalpy changes Δ*H*_exo_ = −156.3 J/g and Δ*H*_endo_ = 247.9 J/g for *m*_4_ compared to Δ*H*_exo_ = −92.1 J/g, and an endothermic enthalpy change Δ*H*_endo_ = 192.8 J/g for *m*_1_. The 69.6% increase in Δ*H*_exo_ and 28.5% rise in Δ*H*_endo_ reflected the influence of sample mass on total energy release, consistent with the cumulative effect of latent heat content in larger samples. The observed trends can be explained by classical nucleation theory (CNT) and thermal transport dynamics [19,20]. As shown in Equation (1), according to CNT, the nucleation energy barrier (expressed per unit mass), $∆G^{*}$, is inversely proportional to the sample volume (*V*):

(1) $$∆G^{*}=\frac{16\pi \gamma^{3}}{3{{\Delta G}_{\nu }}^{2}}\propto \frac{1}{V}$$

where *γ* represents the interfacial energy and $∆G_{\nu }$ denotes the volumetric free energy difference. As the sample volume increases, the nucleation energy barrier decreases, promoting heterogeneous nucleation and leading to more complete crystallization of the *α*-phase. This effect was evident in the 69.6% increase in Δ*H*_exo_ from *m*_1_ to *m*_4_, which corresponded to an enhanced probability of nucleation at interfacial regions due to amplified thermal gradients. In the smaller samples, minimal internal thermal gradients create a uniform temperature field, suppressing heterogeneous nucleation and leading to incomplete crystallization, resulting in lower exothermic enthalpy release [21].

Thermodynamically, the enthalpy change Δ*H* associated with the solid–solid phase transition involves both internal energy and entropy (Δ*S*) contributions, according to the relation Δ*H* = Δ*G* + TΔ*S*. Although Δ*S* cannot be quantitatively determined in this study, its effect on phase stability was implied in the reversible transition behavior observed for AMPD. Based on CNT, the nucleation energy barrier ΔG* determined the possibility of crystallization. While ΔG* was inversely related to the sample volume, larger AMPD samples exhibited enhanced supercooling stability due to a reduced surface-to-volume ratio, which decreased the availability of heterogeneous nucleation sites. This effect prolonged the retention of the metastable *β*-phase in larger systems, consistent with the observed thermal behavior.

A similar trend was observed for endothermic enthalpy that increased with the sample mass, which could be attributed to differences in molecular rearrangement kinetics during reheating [22]. In the smaller samples, where heat is uniformly distributed, the transition from the *α*- to *β*-phase occurs more efficiently, requiring relatively lower energy input. In contrast, the larger samples exhibit spatially inhomogeneous temperature distributions, leading to asynchronous phase transitions across different regions. Some domains require additional thermal energy to fully complete molecular rearrangement and establish the plastic crystalline phase, resulting in a progressive increase in Δ*H*_endo_ with the sample mass. Table S1 presents the exothermic and endothermic enthalpy changes per unit mass for each sample during phase transition. These enthalpy differences reflected the kinetic asymmetry between melting and crystallization, where incomplete nucleation and molecular disorder during cooling led to a lower Δ*H*_exo_ compared to the full enthalpy release during heating.

**Figure 3:**
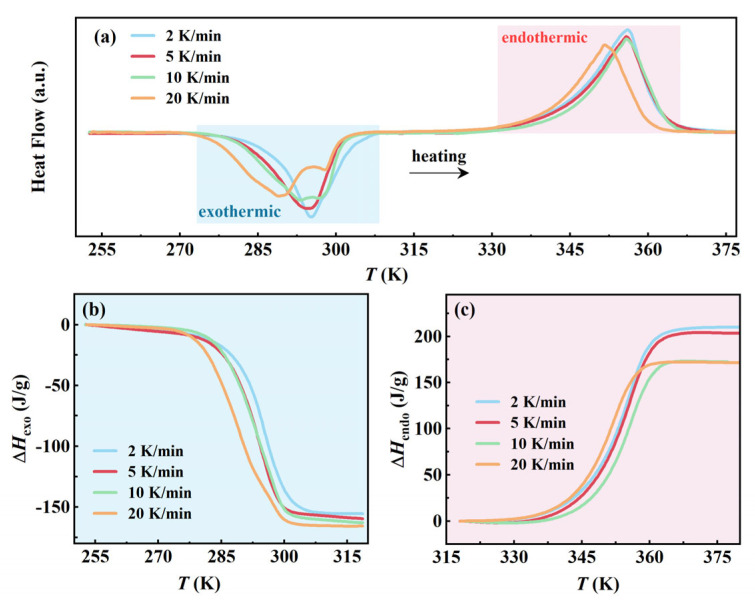
The effect of different cooling rates on the phase transition of AMPD: (**a**) Heat flow curves during heating at a fixed rate of 5 K/min after cooling at different rates, and (**b**,**c**) the variations in exothermic and endothermic enthalpy with temperature during the heating process, respectively.

To elucidate the effect of cooling rate on the phase transition behavior of AMPD, DSC measurements were conducted at the controlled cooling rates of 2, 5, 10, and 20 K/min, followed by a uniform reheating rate of 10 K/min. This experimental design emphasized the influence of cooling rates while maintaining consistent reheating conditions, enabling a direct comparison of the nucleation dynamics and phase stability. Quantitative phase transition parameters are summarized in Table S2. The cooling rate-dependent behavior can be explained through the CNT. As shown in Equation (2), the nucleation rate *J* is expressed as follows:

(2) $$J=J_{0}exp(-\frac{∆G^{*}}{k_{B}T})$$

where *γ* is the interfacial energy and Δ*G_v_* is the volumetric free energy difference between phases [19,20,21]. Increasing the cooling rate from 2 to 20 K/min enhanced supercooling, which elevated Δ*G_v_* (thermodynamic driving force), while reducing the critical nucleation barrier ΔG*. The formed dual effect amplifies stochastic nucleation events, which was consistent with the broadening exothermic peaks and emergent doublet feature at 20 K/min (Figure 3a). Spatial heterogeneity in crystallization, evidenced by the expanded phase transition temperature range Δ*T* from 21.5 K (2 K/min) to 27.8 K (20 K/min), arises from thermal gradients under rapid cooling. These gradients induce localized variations in Δ*G_v_*, promoting heterogeneous nucleation at high-energy interfaces rather than homogeneous bulk crystallization. Concurrently, incomplete molecular alignment during fast cooling generates structural defects in the metastable *β*-phase domains. These defects (e.g., misaligned hydrogen bonds and lattice vacancies) lower the activation energy for the transition from *β-* to *α*-phase by providing pre-existing nucleation pathways. This proposed possible mechanism explains the significant reduction in endothermic enthalpy Δ*H*_endo_ from 209.8 J/g (2 K/min) to 171.4 J/g (20 K/min) (Figure 3c), aligning with defect-mediated transitions in hydrogen-bonded systems [23,24,25].

In contrast to the variable endothermic behavior, the exothermic enthalpy change Δ*H*_exo_ exhibited minimal variation with cooling rates, ranging from −154.9 J/g (2 K/min) to 165.4 J/g (20 K/min) (Figure 3b), which suggested that α-phase crystallization, once nucleated, progresses as a thermodynamically controlled process dominated by equilibrium phase growth rather than kinetic constraints [26]. The phase transition termination temperature *T*_e_ remained constrained within a narrow range from 301.5 to 304.8 K, reflecting the thermodynamic equilibrium boundary of *β*-phase dissociation. The temperature invariance, coupled with the stable Δ*H*_exo_ values, confirmed that the final phase composition is governed by thermodynamic stability thresholds. The decoupling between nucleation onset (*T*_s_ depression) and phase termination (*T*_e_ stability) reflected the interaction between the kinetic constraints and thermodynamic equilibrium in hydrogen-bonded plastic crystals, consistent with classical nucleation theory and phase stability analysis.

**Figure 4:**
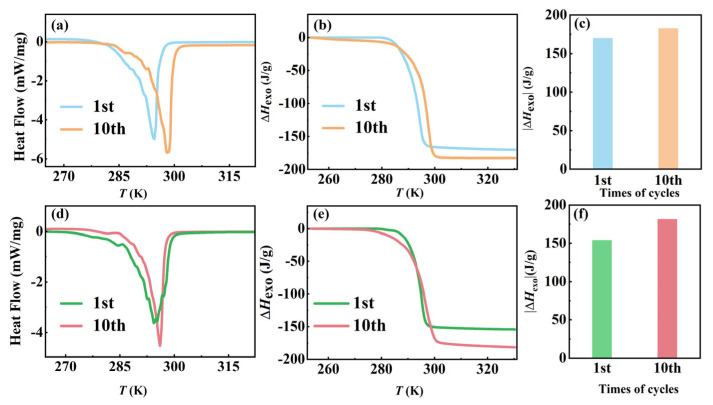
The effect of cooling rates on the exothermic phase transition of AMPD during thermal cycling. (**a**) Heat flow curves and (**b**) enthalpy changes as functions of temperature for a cooling rate of 2 K/min, and (**c**) comparison of the absolute enthalpy changes after ten thermal cycles at 2 K/min. (**d**) Heat flow curves and (**e**) enthalpy changes as functions of temperature for a cooling rate of 10 K/min, and (**f**) comparison of the absolute enthalpy changes after ten thermal cycles at 10 K/min.

To investigate the kinetic–thermodynamic interaction during the crystallization process of AMPD, we performed ten thermal cycles at controlled cooling rates (2 and 10 K/min), with a fixed heating rate of 10 K/min as shown in Figure 4, revealing distinct structural evolution pathways governed by molecular reorganization dynamics. The quantitative phase transition parameters are summarized in Table S3. As shown in Figure 4a–c, for the slow-cooled group (2 K/min), the exothermic enthalpy Δ*H*_exo_ increased progressively from −170.1 to −182.7 J/g over ten cycles, accompanied by a narrow crystallization temperature range from 281.1–297.8 to 284.3–299.1 K. An enhancement of 7.4% in the energy release aligned with the configurational entropy minimization principle in plastic crystals [27], where the prolonged molecular reorganization time reduces lattice defects. The upward shift in the crystallization onset temperature *T*_s_ reflected reduced nucleation barriers due to iterative annealing, which was consistent with the earlier observations on the extended reorganization time enhancing crystallinity in the larger samples. In contrast, as shown in Figure 4d–f, the rapid cooling (10 K/min) initially generated metastable configurations, evidenced by a decreased Δ*H*_exo_ (−154.1 J/g) and broadened crystallization range (277.1–301.6 K). Remarkably, Δ*H*_exo_ was restored to −181.6 J/g after ten cycles (17.8% enhancement), with the range stabilizing at 284.5–300.4 K. The above recovery originates from thermally activated hydrogen-bond reorganization, where the repeated phase transitions eliminate disordered domains through cooperative -OH/-NH_2_ rearrangement, which is analogous to structural relaxation in hydrogen-bonded polymers. Despite the initial differences, both the cooling rates ultimately led to nearly identical thermodynamic states, with a Δ*H*_exo_ difference of less than 1% and a Δ*T* variation below 2%. The results suggested that the slower cooling promotes defect minimization through equilibrium-driven crystallization, while the faster cooling facilitates kinetic recovery via cyclic hydrogen-bond reorganization. This dual-pathway mechanism aligns well with the defect-annealing models in hydrogen-bonded plastic crystals. Furthermore, the enhanced cycling stability of AMPD, compared to the well-known neopentyl glycol system, highlights its structural adaptability, where its hydroxyl-rich framework enables efficient energy dissipation under thermal stress.

**Figure 5:**
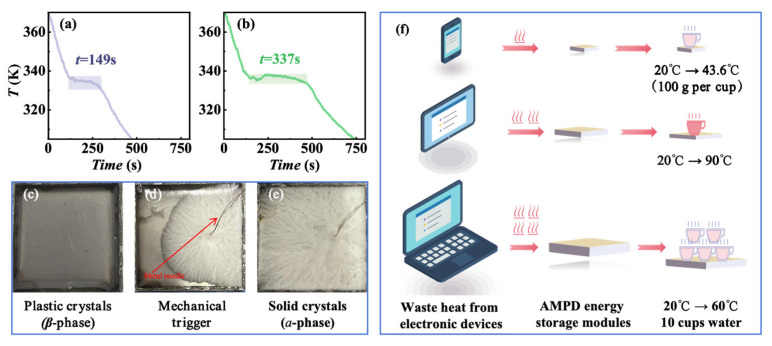
The temperature evolution of AMPD samples during cooling and mechanical triggering. (**a**) *m*_1_ = 1125 mg and (**b**) *m*_2_ = 2250 mg. (**c**) Supercooled plastic crystalline state. (**d**) Phase transition upon triggering at 333.16 K (~60 °C). (**e**) Solid crystalline state after phase transition. (**f**) The application of AMPD in electronic devices (mobile phones, tablets, and laptops) for waste heat recovery, and its secondary utilization for water heating.

An investigation of the phase transition behavior in AMPD revealed a remarkable supercooling persistence, where the high-temperature plastic crystalline *β*-phase remained metastable at room temperature (~298 K) for several months without spontaneous heat release. However, mechanical stimuli (e.g., needle insertion) disrupted the metastability, as shown in Figure S1, triggering rapid heat release. The thermal or mechanical dual-trigger mechanism positions AMPD as a promising candidate for intelligent thermal management systems, particularly in the applications requiring on-demand energy release. To assess the thermal regulation potential of AMPD, a simplified experimental setup was designed using two metal containers (2 cm × 2 cm × 0.5 cm) filled with AMPD samples of different masses (*m*_1_ = 1125 and *m*_2_ = 2250 mg). The samples were first heated to 380 K to ensure complete transition to the *β*-phase, followed by natural cooling to ambient temperature while continuously recording their temperature profiles to track heat dissipation dynamics. In the absence of external intervention, both samples exhibited linear temperature decay (Figure 5a,b), consistent with passive heat dissipation governed by thermal conduction and phase transition kinetics. However, when an external trigger was applied at 333.16 K (60 °C), simulating a thermal regulation threshold, the system behavior changed markedly. Nucleation was initiated near the trigger site, inducing rapid latent heat release and forming distinct temperature plateaus (Figure 5c–e). The plateau durations, 149 s for *m*_1_ and 337 s for *m*_2_, increased with the sample mass, which aligned with the increases in total latent heat, determined by the product of the sample mass and the specific latent heat. This behavior reflected the additive nature of energy storage in phase change systems, rather than the differences in intrinsic material properties. This mechanically triggered phase transition highlights the unique capability of AMPD for active thermal regulation. By strategically timing the external stimuli, the system can maintain target temperatures without continuous energy input, offering an energy-efficient and controllable thermal management solution. These findings confirmed that AMPD enables temporal decoupling between energy storage and release, and supports dual-trigger activation via both thermal input and mechanical stimulation.

Based on the phase transition characteristics and energy storage behavior of AMPD, we developed a modular waste heat recovery system that leverages its high latent heat capacity (Δ*H*_exo_ = −159.5 J/g, averaged across the cooling rates of 2–20 K/min) and mechanically triggered crystallization for efficient thermal energy harvesting and controlled release. The AMPD modules can be directly attached to electronic devices, such as smartphones and tablets, to absorb waste heat through solid–solid phase transitions. In off-grid scenarios, stored thermal energy can be instantaneously released via mechanical triggering to supply warm water (40–60 °C) or hot water (80–100 °C), addressing diverse daily needs. As shown in Figure 5f, to meet diverse thermal management needs, three AMPD modules were designed for standard electronic devices, with the specific parameters detailed in Table S4. The smartphone module (15 × 7.5 × 0.5 cm^3^, 63.4 g), with a thermal storage capacity of 10.11 kJ, was capable of heating 100 g of water from 20 to 43.6 °C for instant warm water supply. The tablet module (21 × 18.6 × 0.5 cm^3^, 220.0 g), with a thermal storage capacity of 35.09 kJ, heated 100 g water from 20 to 90 °C for prolonged heat retention without boiling risks. The laptop module (38 × 25 × 1 cm^3^, 1069.8 g), with a thermal storage capacity of 170.63 kJ, enabled the simultaneous heating of ten 100 g water portions (one cup each) from 20 °C to 60.8 °C, supporting multi-user operation in parallel. Compared to conventional electric kettles (400 W, 5-min heating), AMPD modules eliminated electricity consumption, reducing CO_2_ emissions by 1.5 kg/month (assuming three daily uses and carbon emission factor: 0.5 kg CO_2_/kWh). The plug-and-play design ensures compatibility with diverse electronics, enhancing energy utilization efficiency.

## 3. Materials and Methods

The compound 2-amino-2-methyl-1,3-propanediol (AMPD, purity: 98%) was purchased from Sigma-Aldrich (Shanghai) Trading Co., Ltd. (Shanghai, China) and used without further purification. To eliminate any residual moisture, the sample was thoroughly dried at 60 °C for 24 h in a vacuum oven (0.1 MPa) and then stored in a desiccator to prevent moisture reabsorption. Thermogravimetric analysis (TGA) of the dried sample revealed no significant mass loss below 150 °C, confirming the absence of volatile residues. Additionally, control DSC tests performed on samples dried for 12 and 24 h showed negligible variation (<1.5%) in latent heat and transition temperatures, indicating stable thermal behavior under the selected drying conditions.

The phase transition behavior of AMPD was characterized using differential scanning calorimetry (DSC 4000, PerkinElmer Pyris Diamond, Waltham, MA, USA). All measurements were conducted under a nitrogen atmosphere to minimize oxidation and external environmental influences. The temperature control accuracy of the instrument is ±0.1 °C. The heating and cooling rates were systematically varied between 2 and 20 K/min to examine their effects on phase transition kinetics. The sample was placed in high-purity aluminum oxide crucibles, which were hermetically sealed with lids to prevent possible volatilization during thermal cycling. In terms of the data processing, background correction was performed to eliminate baseline drift and improve measurement accuracy.

## 4. Conclusions

This study systematically investigated the phase transition characteristics of AMPD and its potential in energy storage applications. The results demonstrated that AMPD undergoes a reversible solid-state phase transition during cooling, with its phase transition temperature range and exothermic enthalpy change being influenced by the cooling rate and sample mass. The faster cooling rates suppressed crystal nucleus formation, resulting in a greater supercooling degree and lower exothermic enthalpy change, while the slower cooling rates facilitated molecular ordering and enhanced crystallinity, thereby reducing supercooling and improving the exothermic capacity. Furthermore, the simulated application scenarios confirmed the triggerable exothermic characteristics in AMPD, where external triggers induced the rapid recovery of molecular ordering, generating stable plateau regions during the exothermic process that exhibited excellent thermal stability and controllable energy release. This research reveals that AMPD, as a supercooled phase change energy storage material, demonstrates an interaction mechanism between microscopic molecular rearrangement and adjustable phase transition properties, providing the guidance for its application in thermal management and the energy storage fields.

## Data Availability

The original contributions presented in this study are included in the article/Supplementary Materials. Further inquiries can be directed to the corresponding authors.

## Supplementary Materials

The following supporting information can be downloaded at: https://www.mdpi.com/article/10.3390/molecules30102206/s1, Figure S1. Schematic illustration of the phase transition of AMPD during the heating–cooling–reheating process. During heating from state (a) to state (b), the solid crystalline α-phase undergoes a phase transition into the plastic crystalline β-phase, accompanied by an endothermic enthalpy change. In the subsequent cooling process from state (b) to state (c), the plastic crystalline phase remains in a supercooled state at room temperature due to the absence of spontaneous crystallization. The supercooled plastic crystalline β-phase can revert to the solid crystalline α-phase either through reheating, as observed in the transition from state (c) to state (a), or by mechanical triggering, as demonstrated in state (d), both of which are associated with an exothermic enthalpy change. Table S1. Enthalpy changes during phase transition of AMPD samples with different masses, and Δ*H*_exo_ and Δ*H*_endo_ represent exothermic and endothermic enthalpy changes, respectively; negative values of Δ*H*_exo_ indicate heat release; Table S2. The phase transition parameters of AMPD at different cooling rates, and Ts and Te represent the onset and end temperatures of phase transition, respectively; ΔT is the phase transition temperature range; Table S3. Thermal cycling performance of AMPD at different cooling rates. Table S4. Performance parameters of AMPD modules for different electronic devices.
