# Supplementary material for: Supercooling Behavior of 2-Amino-2-methyl-1,3-propanediol for Thermal Energy Storage
Source: Molecules. 2025 May 18;30(10):2206. doi: 10.3390/molecules30102206 (PMC12114152; doi:10.3390/molecules30102206)
Supplement: Supplementary file 1 [file molecules-30-02206-s001.zip › molecules-3580378-supplementary.pdf]

Supercooling Behavior of 2-Amino-2-Methyl-1,3-Propanediol for Thermal Energy Storage

Xuelian Wang[a], Jin Bai[b,*], Xian Zhang[a,*], Xiaobo Shen[a], Zhengrong Xia[a], and Haijun Yu[a]

[a] School of Electronic Engineering, Huainan Normal University, Huainan 232038, People's Republic of China

[b] Key Laboratory of Materials Physics, Institute of Solid State Physics, The Hefei Institutes of Physical Science (HFIPS), Chinese Academy of Sciences, Hefei 230031, People's Republic of China

***Corresponding Authors**

E-mail address: jbai@issp.ac.cn (J. Bai); zhangxian035@163.com (X. Zhang)

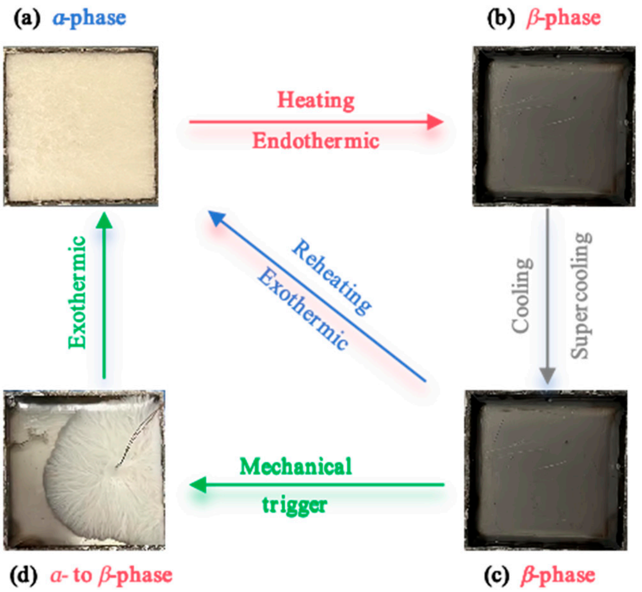

Figure S1. Schematic illustration of the phase transition of AMPD during the heating–cooling–reheating process. During heating from state (a) to state (b), the solid crystalline α-phase undergoes a phase transition into the plastic crystalline β-phase, accompanied by an endothermic enthalpy change. In the subsequent cooling process from state (b) to state (c), the plastic crystalline phase remains in a supercooled state at room temperature due to the absence of spontaneous crystallization. The supercooled plastic crystalline β-phase can revert to the solid crystalline α-phase either through reheating, as observed in the transition from state (c) to state (a), or by mechanical triggering, as demonstrated in state (d), both of which are associated with an exothermic enthalpy change.

Table S1. Enthalpy changes during phase transition of AMPD samples with different masses, and ΔH_{exo} and ΔH_{endo} represent exothermic and endothermic enthalpy changes, respectively; negative values of ΔH_{exo} indicate heat release.

Mass (mg)	m_1	m_2	m_3	m_4
ΔH_{exo} (J/g)	-92.1	-141.7	-153.1	-156.3
ΔH_{endo} (J/g)	192.8	200.6	221.6	247.9

Table S2. The phase transition parameters of AMPD at different cooling rates, and T_s and T_e represent the onset and end temperatures of phase transition, respectively; ΔT is the phase transition temperature range.

Rate (K/min)	T_s (K)	T_e (K)	ΔT (K)	ΔH_{exo} (J/g)	ΔH_{endo} (J/g)
2	283.3	304.8	21.5	-154.9	209.8
5	281.5	303.8	22.3	-157.2	203.7
10	279.1	302.5	23.4	-160.6	171.9
20	273.7	301.5	27.8	-165.4	171.4

Table S3. Thermal cycling performance of AMPD at different cooling rates.

Rate (K/min)	Cycle	T_s (K)	T_e (K)	ΔH_{exo} (J/g)	ΔH_{exo} Change (%)
2	1	281.1	297.8	-170.1	-
2	10	282.5	303.3	-182.7	+7.4
10	1	277.1	301.6	-154.1	-
10	10	284.5	300.4	-181.6	+17.8

Table S4. Performance parameters of AMPD modules for different electronic devices.

Module Type	Dimensions (L×W×H, cm)	AMPD Mass (g)	Heat Release (J)	Water Heating Capacity (1 cup)
Smartphone	15×7.5×0.5	63.4	10112.3	20℃ → 43.6 ℃
Tablet	21×18.6×0.5	220.0	35090.0	20℃ →≥ 90 ℃* （theoretical: 103.9 ℃）
Laptop	38×25×1	1069.8	170633.1	(10 cups): 20 ℃ → 60.8 ℃

Note:

① Heating capacity is calculated based on the heat release of AMPD (ΔH_{exo} = -159.5 J/g) and the specific heat capacity of water (4.18 J/g·K). One cup of water is assumed to be 100 g.

② Theoretical heating to 103.9 °C exceeds the boiling point of water (100 °C at 1 atm). To avoid boiling risks, the temperature is capped at 90.0 °C for practical applications.
